# Immune physiology in tissue regeneration and aging, tumor growth, and regenerative medicine

**DOI:** 10.18632/aging.100024

**Published:** 2009-02-13

**Authors:** Antonin Bukovsky, Michael R. Caudle, Ray J. Carson, Francisco Gaytán, Mahmoud Huleihel, Andrea Kruse, Heide Schatten, Carlos M. Telleria

**Affiliations:** ^1^ Laboratory of Development, Differentiation and Cancer, Department of Obstetrics and Gynecology, The University of Tennessee College of Medicine and Graduate School of Medicine, Knoxville, TN 37920, USA; ^2^Department of Medical & Social Care Education, Leicester Medical School, University of Leicester, Leicester, LE1 9HN, UK; ^3^University of Cordoba, Department of Cell Biology, Physiology and Immunology, Cordoba, 14004, Spain; ^4^The Shraga Segal Department of Microbiology and Immunology, Beer-Sheva, 84105, Israel; ^5^Institute of Systemic Inflammation Research, University of Luebeck, Luebeck, 23538, Germany; ^6^University of Missouri; Columbia, MO 65211, USA; ^7^Division of Basic Biomedical Sciences, Sanford School of Medicine of The University of South Dakota, Vermillion, SD 57069, USA

**Keywords:** immune physiology, tissue homeostasis, mesenchymal-epithelial interactions, proliferation, regeneration, aging, follicular renewal, animal models, tumor growth, regenerative medicine

## Abstract

The immune system plays an important role in immunity (immune surveillance), but also in the regulation of tissue homeostasis (immune physiology). Lessons from the female reproductive tract indicate that immune system related cells, such as intraepithelial T cells and monocyte-derived cells (MDC) in stratified epithelium, interact amongst themselves and degenerate whereas epithelial cells proliferate and differentiate. In adult ovaries, MDC and T cells are present during oocyte renewal from ovarian stem cells. Activated MDC are also associated with follicular development and atresia, and corpus luteum differentiation. Corpus luteum demise resembles rejection of a graft since it is attended by a massive influx of MDC and T cells resulting in parenchymal and vascular regression. Vascular pericytes play important roles in immune physiology, and their activities (including secretion of the Thy-1 differentiation protein) can be regulated by vascular autonomic innervation. In tumors, MDC regulate proliferation of neoplastic cells and angiogenesis. Tumor infiltrating T cells die among malignant cells. Alterations of immune physiology can result in pathology, such as autoimmune, metabolic, and degenerative diseases, but also in infertility and intrauterine growth retardation, fetal morbidity and mortality. Animal experiments indicate that modification of tissue differentiation (retardation or acceleration) during immune adaptation can cause malfunction (persistent immaturity or premature aging) of such tissue during adulthood. Thus successful stem cell therapy will depend on immune physiology in targeted tissues. From this point of view, regenerative medicine is more likely to be successful in acute rather than chronic tissue disorders.

## Introduction

More than eighty years ago, Alexis Carrel demonstrated that leukocyte extracts, like embryonic tissue extracts, stimulate multiplication of fibroblasts *in vitro*, and suggested that leukocytes can bring growth-activating substances to tissue-specific cells [1]. More recently, lymphocytes and also monocyte derived cells (MDC) were shown to promote tissue growth and regeneration [2-7].

It has been suggested that only one of the many functions of lymphocytes is their participation in host immune responses since lymphoid cells as "trephocytes" also participate in a number of physiological processes aimed at maintaining homeostasis [2]. In addition, abundance of tumor-associated macrophages is correlated with poor prognosis. It has been hypothesized that besides normal trophic functions, the MDC promote tumor progression and metastasis [8]. However, while a lot of work has been done to examine the influence of various growth factors and cytokines produced by lymphocytes and MDC on the cell cycle and death *in vitro* [9-14], little is known about interactions between these immune system-related mesenchymal cells and tissue-specific cells *in vivo*.

The biological role of intraepithelial immune system-related cells has remained an enigma to researchers for many years. It is still widely believed that the only role of MDC and gamma delta T cells in epithelial tissues such as the skin, gut, and lung is in maintaining tissue integrity, defending against pathogens, regulating inflammation, wound healing, and monitoring neighboring cells for signs of damage or disease [5,6,15,16]. However, there are tissues which do not communicate with the outer environment, such as the ovarian corpus luteum (CL), in which MDC and T cells accompany differentiation and demise of epithelial cells [3,17,18]. Therefore, we hypothesize that the primary role of intraepithelial MDC and T cells is to maintain tissue homeostasis, such as proliferation, differentiation, and preservation of epithelial or parenchymal cells in a functional state (immune physiology).

Alteration of immune physiology can by itself cause alteration of tissue function (immune pathology), such as rheumatic [19] and degenerative diseases [20]. Secondarily, if needed, the MDC and T cells are converted into effectors of immunity defending against pathogens (immune surveillance). Moreover, the role of immune system components in the regulation of tissue physiology and pathology should be viewed in context with resident mesenchymal cells, such as vascular pericytes derived from stromal fibroblasts, as well as neuronal signals.

This review article focuses on the role of immune system-related cells and molecules they produce in the regulation of epithelial and parenchymal cell proliferation, differentiation, and aging, and describes a theory of the so called Tissue Control System (see below) in the reproductive tract, which may be universal for other tissues as well. Some implications of immune physiology for augmentation of cancer and efficient utilization of regenerative medicine are also suggested.

### THE TISSUE CONTROL SYSTEM THEORY

To study the role of immune physiology in homeostasis of tissues in general, the tissues with fast cellular development and demise are essential. The female reproductive tract tissues represent one of the most dynamic and active structures within the mammalian body. Our studies in the late 1970s [21-24] and early 1980s [25,26] resulted in the concept of a wider role of the immune system (immune system cells and vascular pericytes), the so called Tissue Control System (TCS), in regulation of ovarian function [27]. The TCS theory was further refined when the role of autonomic innervation in the regulation of quantitative aspects in tissues, including ovarian follicular selection, was added, [28,29] and the TCS theory was revised [30,31]. Autonomic innervation plays an important role in determination of the extent of tissue development since an elimination of limited areas of the cephalic neural crest in stage 9 or 10 chick embryos markedly reduced the size of the thymus gland or resulted in its absence. Small thymic lobes contained both normal thymocytes and epithelial cells, but showed delayed development [32]. More recently, a role for the immune system-related cells in the regulation of ovarian aging [33,34] and regulation of asymmetric cell division of germ cell progenitors, giving rise to new germ cells during the fetal period and adulthood [35-37], have been described.

### Basic "tissue control unit" and "immune physiology"

The TCS consists of immune system-related cells (MDC and T and B lymphocytes), vascular pericytes, and autonomic innervation. While immune reactions are directed against foreign substances (immune surveillance), the TCS is proposed to regulate regeneration, preservation and aging of tissue specific cells ("immune physiology") including the female reproductive tissues [27,30,33]. Ovary, uterus and, in the case of pregnancy, the placenta exhibit periodic growth and regression, which are extremely rapid and are accompanied by changes in rates of blood flow. Therefore, it is not surprising that angiogenesis and remodeling of the local epithelium and vascular bed occur as a normal process in these tissues [38-41].

**Figure 1.**
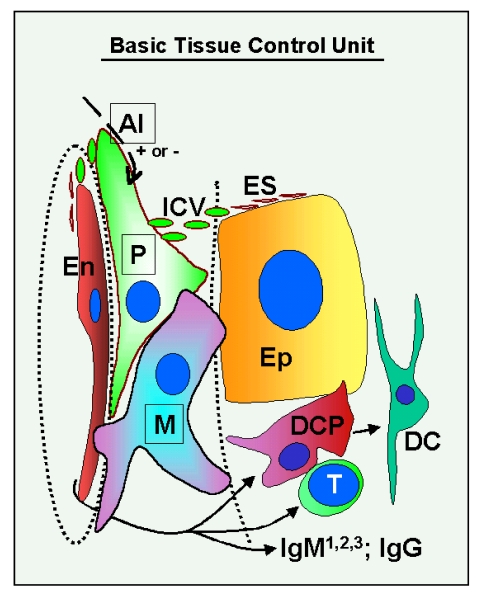
Schematic drawing of the basic "tissue control unit," which consists of monocyte-derived cells (marked M in the figure), vascular pericytes (P), and autonomic innervation (AI, dashed arrow), and the involvement of other components of the tissue control system (solid arrows). Monocyte-derived cells physically interact with adjacent epithelial (Ep) and endothelial cells (En) through the basement membranes (dotted lines), and influence pericytes, which secrete intercellular vesicles (ICV). These vesicles collapse into the so-called empty spikes (ES) releasing their content (growth factor/cytokine) after reaching target cells. The activity of pericytes is stimulated or inhibited by autonomic innervation (+ or -) which controls quantitative aspects of tissues. Interaction of MDC with endothelial cells may stimulate homing of T lymphocytes (T) and monocyte-derived dendritic cell precursors (DCP; also known as veiled cells) differentiating into mature dendritic cells (DC). The dendritic cell precursors and T cells interact themselves and stimulate advanced differrentiation of epithelial cells. IgMs regulate early (IgM1), mid (IgM2), and late differentiation (apoptosis) of epithelial cells (IgM3), and IgG associates with aged cells (see Figure 2 and 3). The monocyte-derived cell system (including intraepithelial DCP and mature DC) is postulated to play a dominant role in the regulation of qualitative aspects of tissue-specific cells, including expression of ligands for intraepithelial T cells and regulating autoantibody action. Monocyte-derived cells also carry "stop effect" information (Figure 10B), presumptively encoded at the termination of immune adaptation (Figure 10A), which determines the highest state of epithelial cell differentiation allowed for a particular tissue. For details see Ref. [3,4,33]. Reprinted from Ref. [4], © Antonin Bukovsky.

The basic "tissue control unit" (TCU) is associated with tissue microvasculature. Monocyte-derived cells (marked M in Figure 1) interact with vascular pericytes (P), and both cell types regulate, via growth factors and cytokines, proliferation, differentiation, and apoptosis of tissue specific epithelial (Ep) and endothelial cells (En). The influence of TCU on endothelial cells plays an important role in the control of homing of tissue-committed circulating MDC and T cells, a process which is mediated by highly regulated vascular adhesion molecules and by chemoattractant factors. The intraepithelial MDC [dendritic cell precursors (DCP) and dendritic cells (DC)], T cells (T), and natural autoantibodies (three types of IgM: IgM1, IgM2 and IgM3 - see later, and one type of IgG) [4] play an important role in the control of **qualitative** aspects (differentiation and aging) of tissue cells, and autonomic innervation controls **quantitative** aspects of tissues by regulation of TCU activity [AI (+ or -)] [3,4].

### Complete TCS pathway reflects immune system phylogeny

**Figure 2.**
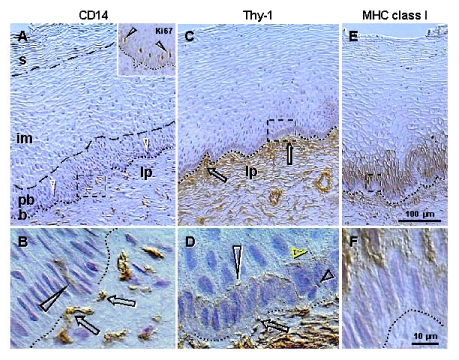
Peroxidase immunohistochemistry (brown color) of stratified epithelium of uterine ectocervix as indicated above columns and in the inset. (**A**) CD14 primitive MDC in lamina propria (lp) associate with the epithelium basement membrane (dotted line). Dashed box indicates detail shown in (**B**). **b**, basal layer; **pb**, parabasal layer; **im**, intermediate layer; **s**, superficial layer. Arrowheads, basal/parabasal interface; dashed line, parabasal/intermediate interface; dashed/dotted line, intermediate/superficial interface. Ki67 staining (inset) of epithelial cells in lower parabasal layer (arrowheads). (**B**) CD14 MDC (arrows) exhibit extensions among basal cells (arrowhead). (**C**) Pericytes of microvasculature (arrows) associate with the basement membrane. (**D**) Detail from (C) shows intercellular Thy-1 vesicles (arrow) secreted by pericytes and migrating among basal cells (short black arrowhead) to basal/parabasal interface (long arrowhead). Yellow arrowhead indicates residual empty structures ("spikes"). (**E**) Strong MHC class I expression (W6/32 antibody specific for heavy chain) is characteristic of para-basal cells, and diminishes in lower intermediate layers. Dashed box indicates detail shown in (**F**).** (F**) Basal cells show no MHC class I expression. Reprinted from Ref. [4], © Antonin Bukovsky.

Examples of complete TCS involvement in the regulation of cellular differentiation, from the stem to mature and aged cells, can be found in some stratified epithelial tissues, such as uterine ectocervix. The stratified epithelium of uterine ectocervix consists of four layers of epithelial cells, basal (b), parabasal (pb), intermediate (im), and superficial cells (s; see Figure 2A). The basal layer is formed by a single row of basal or stem cells. The parabasal layer contains several layers of parabasal (young) epithelial cells, the intermediate layer consists of multiple layers of mature epithelial cells, and the superficial layer is formed by several layers of aged cells. These four morphologically distinct layers are divided by three interfaces - b/pb, pb/im, and im/s interface. The parabasal and intermediate layers can be divided into the lower, mid, and upper layers, the superficial layer into the lower and upper layers.

Mesenchymal cells are present in the lamina propria and invade among epithelial cells. Staining for CD14 of primitive MDC (Figure 2A) shows small MDC in the lamina propria but not within the epithelium. Figure 2B, shows association of CD14 cells with the basement membrane (arrows) and extension among basal epithelial cells (arrowhead). This indicates that primitive MDC may stimulate proliferation of stem cells. The CD14 is a lipopolysaccharide receptor, and is involved in the stimulation of cell proliferation [42]. Similar association of CD14 primitive MDC with proliferating epithelial cells was detected in ovarian cancers (see later). Staining for Ki67 (inset, Figure 2A) shows that this marker of proliferation is expressed in the nuclei of parabasal cells adjacent to the b/pb interface. This indicates that in the stratified epithelium of ectocervix, Ki67 is expressed in postmitotic cells leaving the basal layer and beginning differentiation.

These observations indicate that primitive MDC accompany proliferation of basal epithelial (stem) cells.

### Recognition at the cell surface

Most of the molecules involved in the TCS pathway belong to the immunoglobulin (Ig) superfamily of molecules. It has been suggested that the involvement of Ig-related molecules in tissue interactions is more primitive than their involvement in the immune system and the immune functions evolved from the sets of molecules mediating tissue interactions [43]. One of them, the Thy-1 differentiation protein, consists of a single Ig domain and represents the most primitive and ancestral member of the Ig-superfamily. The Ig-related molecules have a diversity of functions, but in most cases the common denominator is recognition at the cell surface [44]. Also, the only function of Thy-1 differentiation protein and other Ig-related molecules is to mediate recognition, with the consequences of recognition being due to the differentiated state of the cells. It requires that the correct ligand and receptor are expressed on the appropriate cells at the right time [43].

Staining for Thy-1 differentiation protein (Figure 2C) shows pericytes associated with microvasculature (arrows) adjacent to the basement membrane. Detail of Thy-1 staining (Figure 2D) shows that pericytes secrete intercellular vesicles, which migrate among basal epithelial cells to the b/pb interface, where they collapse into empty structures ("spikes"). Hence, targets for Thy-1 vesicles appear to be parabasal cells adjacent to the b/pb interface, i.e., epithelial cells expressing Ki67 and entering differentiation.

These intercellular Thy-1 vesicles have been shown by immunoelectron microscopy to exhibit Thy-1 surface expression and to contain a substance lacking Thy-1 staining [30]. They may represent a unique paracrine mechanism, so called "targeted delivery," by which certain growth factors (vesicle contents) are delivered to certain type/stage specific target cells expressing receptor for Thy-1 ligand. However, the receptor for Thy-1 has not yet been identified. One possibility is that the Ki67+ cells entering differentiation are the targets for Thy-1+ intercellular vesicles. Also, there is a lack of expression of major histocompatibility complex (MHC) class I molecules in epithelial cells adjacent to the basement membrane, but strong staining in parabasal cells (Figure 2E and F). Hence, MHC class I molecules could be involved in the recognition of the Thy-1 ligand.

Targeted delivery of some tissue non-specific (stimulating many types of tissues) growth factors to particular tissue cells by intercellular Thy-1 vesicles could be enabled by tissue specificity of Thy-1 glycoprotein carbohydrate moieties [45].

**Figure 3.**
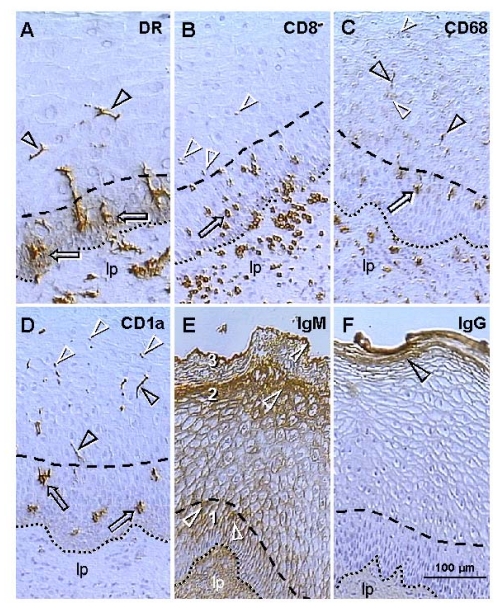
Uterine ectocervix immunohistochemistry as indicated above columns. (**A**) Dendritic cell (DC) precursors secrete HLA-DR among parabasal cells (arrows) and differentiate into mature DC (arrowheads). (**B**) T cells migrate through parabasal layer (arrow) to parabasal/intermediate interface (dashed line) and show fragmentation after entering the intermediate layer (arrowheads). (**C**) Transformation of DC precursors into mature DC at the top of parabasal layer is associated with CD68 expression (arrow). Mature DC (black arrowheads) secrete CD68 material in intermediate layer accompanying mature (intermediate) and aged (superficial) epithelial cells (white arrowheads). (**D**) CD1a is expressed by DC precursors (arrows) and mature DC (black arrowheads). Mature DC (Langerhans' cells) undergo fragmentation in the mid intermediate layer (white arrowheads). (**E**) Strong IgM binding (arrowheads) in upper parabasal [1], upper intermediate [2] and upper superficial layers [3]. (**F**) IgG binds to the entire superficial layer. For abbreviations see Figure 2. Reprinted from Ref. [4], © Antonin Bukovsky.

MHC class I and class II molecules are other Ig superfamily members. Figure 3A shows that large quantities of HLA-DR molecules are secreted by precursors of dendritic cells among epithelial cells in the mid parabasal layer (arrows). This site-specific HLA-DR secretion is particularly evident when DC precursors are compared with inactive MDC in the lamina propria (lp) or mature DC in intermediate epithelial layers (arrowheads).

These observations indicate that recognition at the cell surface by Ig superfamily members may play an important role in immune physiology.

### Degeneration of intraepithelial T cells and MDC and differentiation of epithelial cells

T cells expressing CD8, which is another member of the Ig superfamily, accumulate in the lamina propria, enter the epithelium, and migrate through the parabasal layers (arrow, Figure 3B), toward the pb/im interface (dashed line). T cells entering lower intermediate layers exhibit fragmentation (arrowheads). No T cells were detected in the mid intermediate layers or at the epithelial surface.

The CD68 epitope of mature intraepithelial MDC, a mucin-like molecule belonging to the lysosomal-associated membrane protein family [46], is expressed by MDC in the lamina propria (Figure 3C). However, within epithelium, CD68 appears during transformation of DC precursors into mature DC, in the upper parabasal layers (arrow). Mature DC (black arrowheads) secrete CD68 among intermediate epithelial cells, and CD68 mucin-like molecules accompany advanced differentiation of epithelial cells (white arrowheads) including aging in surface layers.

Staining for CD1a, an Ig-related molecule characteristic for intraepithelial Langerhans' cells, was not detected in the lamina propria (Figure 3D). Dendritic cell precursors (arrows) and mature DC (black arrowheads) were stained. Mature DC reaching mid intermediate layers exhibited fragmentation (white arrowheads) similar to that of T cells in the lower intermediate layers.

It is apparent that intraepithelial T cells crossing the pb/im interface degenerate (Figure 3B), and such apoptosis may be required for a release of substances enabling maturation of epithelial cells. In addition, Figure 2A, 3A, and 3C and D, show that intraepithelial MDC also exhibit morphological and immunohistochemical features accompanying their maturation and demise.

These observations suggest that degeneration of intraepithelial T cells and MDC may be required for advanced differentiation of epithelial cells.

### Association of natural IgMs and IgG with epithelial differentiation

Natural autoantibodies are present in the blood of normal healthy individuals, and they are almost exclusively IgM antibodies, although some IgG and IgA natural autoantibodies can also be detected, that bind to a variety of self-antigens, including self IgG [47,48]. When compared to IgG, the IgM molecules appear earlier in phylogeny and ontogeny [49,50].

Staining of ectocervical epithelium for IgM is shown in Figure 3E. Basal and lower parabasal layers are unstained, but IgM binding increases toward the pb/im interface (#1). In the intermediate layers, a similar increase of IgM binding is apparent toward the im/s interface (#2). In the superficial layers the most prominent staining is evident at the epithelial surface (#3). Hence, there is high IgM binding to the upper cells in the parabasal, intermediate, and surface layers (white arrowheads). IgG does not bind to the basal, parabasal or intermediate cells, but shows binding to the entire superficial layer (arrowhead, Figure 3F).

These data indicate that natural autoantibodies exhibit a stage-specific (differentiation-dependent) binding to epithelial cells. Similar stage-specific binding to epidermis was also detected for natural IgM and IgG autoantibodies in normal human sera [51].

### Interaction of intraepithelial T cells and MDC and T cell demise

**Figure 4.**
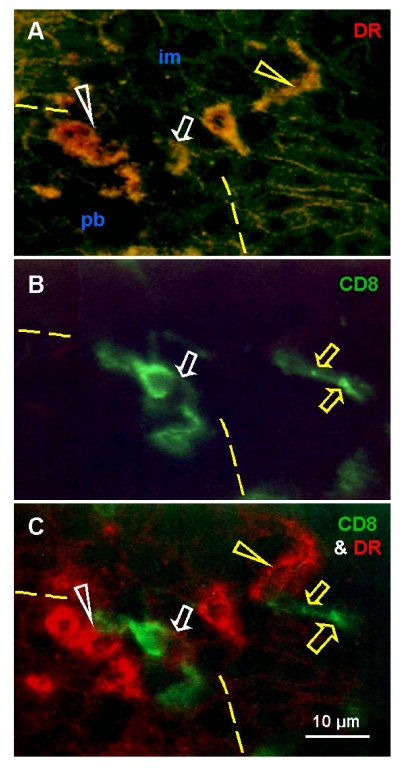
Uterine cervix dual color immunohistochemistry (HLA-DR peroxidase/CD8 FITC) viewed in dark field visible light (**A**), incident fluorescence (**B**) and dark field fluorescence (**C**). (**A**) Interface (dashed line) between parabasal and intermediate layers. White arrowhead shows differentiating DC, yellow arrowhead shows mature DC. Arrow indicates activated T cell with HLA-DR expression (see below). (**B**) White arrow indicates T cell exhibiting unusual elongated shape at the interface. Yellow arrows indicate residual CD8 expression in fragmented T cell among adjacent im epithelial cells. (**C**) Activated T cell with HLA-DR expression (white arrow) interacts with differentiating DC (white arrowhead). Mature DC (yellow arrowhead) accompany T cell fragmentation (yellow arrows). Reprinted from Ref. [4], © Antonin Bukovsky.

Basal and parabasal layers of normal ectocervical epithelium show the presence of T cells and MDC. A possibility exists that, beside interaction with epithelial cells, these mesenchymal cell types may interact with each other. Figure 4 shows the pb/im interface in detail, with staining for HLA-DR MDC (A), CD8 for T cells (B) and both (C). T cells appear to assist differentiation of DC (white arrowheads) and exhibit an unusual elongated shape accompanied by HLA-DR expression (white arrows). Above this interface, the mature DC (yellow arrowhead) accompany fragmentation of T cells (yellow arrows).

These data suggest that transition of parabasal into the intermediate epithelial cells at the pb/im interface is associated with transformation of DC precursors into mature DC with the assistance of activated (HLA-DR+) T cells. The T cells entering intermediate layers show a loss of HLA-DR expression and undergo fragmentation and demise with the assistance of mature DC.

### LESSONS FROM MAMMALIAN FEMALE REPRODUCTION

Mammalian female reproduction is much more complex when compared to males and non-mammalian females. Gonads of adult males contain germ cells (spermatogonia), which produce fresh gametes. Such germ cells are, however, not present in adult female gonads of higher vertebrates, including mammals. Because of that, a dogma evolved about fifty years ago that the process of oogenesis in the animal kingdom follows a uniform pattern, of which there are two main variants. One variant is that the oogenesis appears to continue either uninterruptedly or cyclically throughout reproductive life - e.g. most teleosts, all amphibians, most reptiles and relatively few mammals. The other variant is that the oogenesis occurs only in fetal gonads, and oogonia neither persist nor divide mitotically during sexual maturity - e.g. cyclostomes, elasmobranchs, a few teleosts, perhaps some reptiles, all birds, monotremes, and with a few possible exceptions, all eutherian mammals [52,53]. Nevertheless, in the early 1970s, this belief was felt unwarranted due to a lack of detailed study of adult mammalian ovaries. A thorough reexamination of oogenesis, using modern techniques at well-defined stages of the reproductive cycle, was suggested [54].

In addition, it is also currently believed that oogonia in fetal ovaries of higher vertebrates originate from primordial germ cells, which differentiate into oogonia producing definitive oocytes. However, it is apparent that germ cells are present at the ovarian surface of midpregnancy human fetuses [55]. Our observations support the view that primordial germ cells play a role in the commitment of the surface ovarian stem cells (OSC) toward production of secondary germ cells, and then degenerate (reviewed in [56]). Secondary germ cells are formed in fetal and adult human ovaries by asymmetric division of OSC, with the assistance of MDC and T cells [35,36,57].

Mammalian ovarian compartments belong to those structures showing most pronounced morphological (cellular proliferation, differentiation and regression) and functional changes within the body. Regulation of ovarian function is quite complex, involving interactions between follicular compartments (oocyte, granulosa, and theca cells), as well as the influence of sex steroids produced by follicles, CL and interstitial glands originating from the theca of degenerating follicles. Additionally, communication of the ovary with the hypothalamo-pituitary system and the influence of gonadotropins, autonomic innervation, growth factors and cytokines produced by mesenchymal cells derived from the immune system, all regulate functions of ovarian compartments. While gonadotropins are essential for follicular maturation and ovulation [58], autonomic innervation is necessary for the regulation of follicular selection [59,60].

Interactions between the immune system and ovary are numerous, as immune cells are associated with regulation at every level of the hypothalamo-pituitary-ovarian axis, regulating growth and regression of both follicles and CL [61-63].

### Oogenesis in fetal and adult human ovaries

Earlier observations indicated that secondary germ cells develop in fetal ovaries from the somatic OSC, i.e. "germinal" (surface) epithelium of the ovary [64]. However, until the work of Dustin [65], there were no questions regarding the fate of primordial germ cells within developing ovaries. He recognized two kinds of cells in the germ-line history of amphibians: [1] primordial germ cells, which populated the developing gonad, differentiated into gonocytes, and degenerated, and [2] secondary germ cells originating from the ovarian "germinal" epithelium, which differentiated into definitive oocytes.

Rubaschkin [66] suggested division of the history of the germ cell route (Keimbahn) into three periods. The first period begins with the differentiation of primordial germ cells, which, however, do not have a perspective to become definitive gametes (Urgeschlechtszellen). The gonadal development is associated with the establishment of the so called germinal epithelium (Keimepithel). The second period is associated with the appearance of female or male sex specific cells (Ureier or Ursamenzellen). The third period deals with the development of the sex-specific glands.

The germ cell route of Rubaschkin again raised a question of the fate of primordial germ cells [67]. Winiwarter and Sainmont [68] suggested that these cells degenerate after reaching the sex gland, and that definitive germ cells arise from the ovarian "germinal" epithelium.

**Figure 5.**
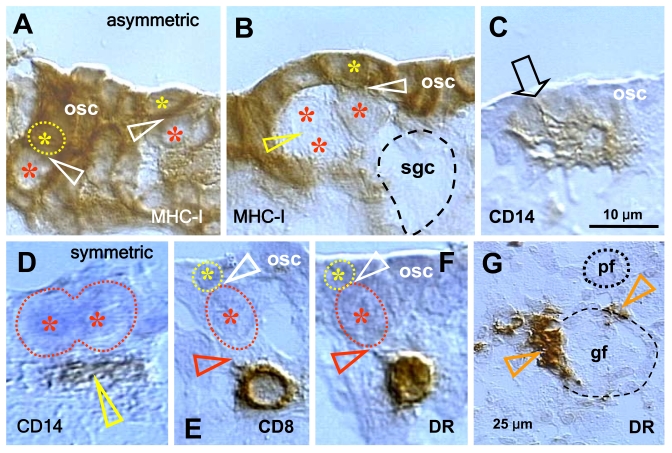
Expression of MHC class I heavy chain (MHC-I), CD14 of primitive MDC, CD8 of T cells, and HLA-DR (DR) of activated MDC and T cells, as indicated in the panels, in human fetal ovary obtained at midpregnancy (24 weeks). Asymmetric division (white arrowheads, panels **A** and **B**) of OSC (osc) gives rise to the OSC (yellow asterisks) and the germ cell daughters (red asterisks). Symmetric division of germ cells follows (yellow arrowhead, panel **B**), which is required for crossing over, and the secondary germ cells (sgc) attain the ameboid shape (dashed line, no hematoxylin counterstain) to leave the OSC layer and enter cortex. CD14+ primitive MDC interact with the OSC (arrow, panel **C**) and accompany (arrowhead, panel **D**) symmetric division of secondary germ cells. CD8 T cells (panel **E**) and DR+ cells of lymphocyte type (panel **F**) accompany (red arrowheads) asymmetric division of OSC (white arrowheads) resulting in emergence of secondary germ cells. DR+ MDC (arrowheads, panel **G**) associate with growing (gf) but not resting primordial follicles (pf). Bar in **C** for **A-F**. Adapted from Ref. [36], © Humana Press.

Our observations indicate that secondary germ cell development in midpregnant human fetal ovary is triggered by MDC and T cells (Figure 5). The germ cells are depleted of major histocompatibility complex class I (MHC-I) expression (red asterisks, Figure 5A and B), and they originate by asymmetric division (white arrowheads) from OSC densely expressing MHC-I (yellow asterisks). A symmetric division of the germ cells, required for crossing over of chromosomes [69], follows (yellow arrowhead, panel B). The germ cells (gc) take on an ameboid shape (dashed line, no hematoxylin counterstain), and enter the adjacent ovarian cortex. Primitive CD14-expressing MDC interact with OSC (arrowhead, panel C), and accompany (arrowhead, panel D) subsequent symmetric division of the germ cells (asterisks). T cells expressing CD8 (panel E) and showing activation (HLA-DR expression, panel F) accompany (black arrowheads) asymmetric division of OSC (white arrowheads, panels E and F). Note that during asymmetric division the emerging germ cells daughters (red asterisks) are substantially larger than OSC daughter cells (yellow asterisks). The activated (HLA-DR+) MDC are associated with growing (gf and arrowheads, panel G) but not resting primordial follicles (pf) [36,70].

Similar emergence of secondary germ cells by asymmetric division of OSC triggered by MDC and T cells has been observed in adult human ovaries [35,57] and expression of a meiosis marker, the meiotic entry synaptonemal complex protein-3 (SCP3), in segments of tunica albuginea and OSC, and in some oocytes of primordial follicles was detected in functional adult human and monkey ovaries [71]. In addition, we also have shown emergence of secondary germ cells by asymmetric division of OSC triggered by MDC and T cells in adult rat ovaries [72]. This indicates that MDC and T cells induce emergence of secondary germ cells from the OSC in various mammalian species.

**Figure 6.**
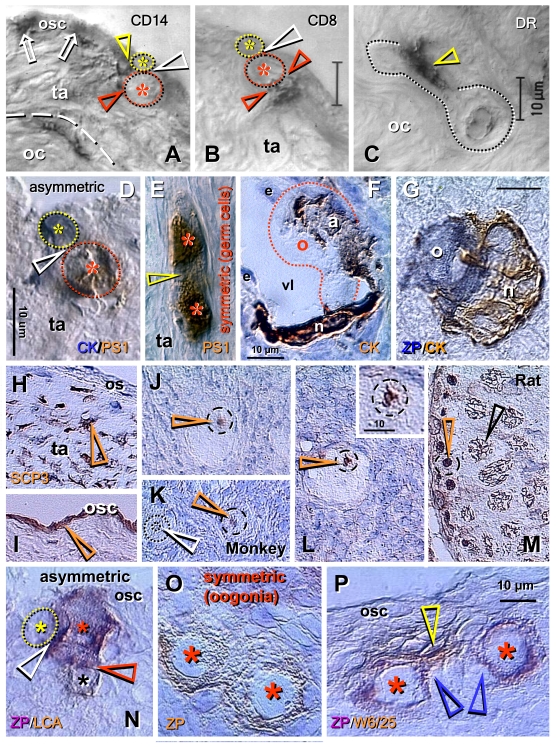
Origin of new oocytes (neo-oogenesis), primordial follicles, and SCP3 expression in adult human and monkey ovaries (**A-M**), and oogenesis in adult rat ovaries (**N-P**). (**A**) During asymmetric division (white arrowhead), the CD14 MDC interact with both the OSC daughter (yellow arrowhead) and germ cell daughter (red arrowhead). (**B**) T lymphocytes, however, interact with the germ cell daughter only (red arrowheads). (**C**) Ameboid germ cells (dotted line) migrating through the dense ovarian cortex (oc) are accompanied by activated MDC (arrowhead). (**D**) Asymmetrically dividing OSC produce a new PS1+ germ cell (red asterisk) and CK+ progenitor cell (yellow asterisk). (**E**) In the tunica albuginea (ta) germ cells (asterisks) symmetrically divide (arrowhead). (**F**) Capture of oocyte (o) from the blood circulation by an arm (a) of granulosa cell nest (n) lining the venule lumen (vl); e, endothelial cells. (**G**) Oocyte nest assembly. (**H**) Segments of tunica albuginea (ta) in ovaries with follicular renewal (early luteal phase) showed strong SCP3 expression of mesenchymal (arrowheads) OSC precursors under ovarian surface (os). (**I**) Staining of OSC (osc and arrowhead) was apparent in other segments - note lack of staining of tunica albuginea under developed OSC. (**J**) Postovulatory human ovaries showed staining of oocyte nucleoli (arrowhead) in some primordial follicles. (**K**) In monkey ovaries, similar staining of oocyte nucleoli in some primordial follicles was observed (red vs. white arrowhead). (**L**) Staining of paired chromosomes oocyte was observed in human ovaries (inset shows higher magnification). (**M**) Adult rat testis (positive control) showed staining of condensed chromosomes in spermatogonia (red arrowhead) and progression of meiotic division in primary spermatocytes (black arrowhead). Oogenesis in adult rat ovaries is initiated by asymmetric division of OSC (white arrowhead, **N**) showing unstained OSC daughter (yellow asterisk) and ZP+ (magenta color) germ cell daughter (red asterisk) accompanied like in human ovaries by a lymphocyte (black asterisk and brown color). Symmetric division of ZP+ oogonia (asterisks, **O**) follows, and is accompanied (**P**) by MDC (yellow arrowhead). Blue arrowheads in (**P**) indicate association of primitive granulosa cells with this process. ZP, zona pellucida; LCA, leukocyte common antigen; W6/25, marker of rat MDC. Details in text. Adapted **A-C**    from Ref. [57], © Blackwell Munksgaard, **D-G**    from Ref. [35], © Antonin Bukovsky, **H-M**    from Ref. [71], © Landes Bioscience, **N-P**    from Ref. [72], © Landes Bioscience.

Adult human ovaries exhibiting neo-oogenesis showed association of CD14 primitive MDC with OSC (arrows, Figure 6A). During asymmetric division, both the emerging germ cell daughter (red asterisk) and the OSC daughter cells (yellow asterisk) were accompanied by extensions of CD14 MDC (see color matching arrowheads). It is apparent, however, that interaction of CD8 T cells is unique for the emerging germ cells (red arrowheads, Figure 6B), see also Figure 5E and F. This suggests that the number of interacting CD8 T cells may determine the number of emerging secondary germ cells in fetal and adult human ovaries. As in fetal ovaries, asymmetric division resulted in the emerging germ cell daughter being larger than the OSC daughter. The HLA-DR (activated) MDC accompanied (arrowhead, Figure 6C) migration of germ cells exhibiting ameboid shape through the dense upper ovarian cortex toward ovarian vessels, which they entered [57].

Dual color immunohistochemistry has shown that during asymmetric division OSC daughters retain cytokeratin expression (blue color, Figure 6D), but the emerging secondary germ cell loses it and attains expression of meiotically expressed PS1 carbohydrate (brown color) [73,74]. As in fetal ovaries, symmetric division of secondary germ cells follows (Figure 6E). The secondary germ cells entering ovarian venules enlarge to the size of small oocytes and are caught in the deep ovarian cortex by the arms (a, Figure 6F) of primitive granulosa cell nests (n) lining the venule lumen (vl). More advanced nest-oocyte assembly resembling an occupied bird's nest is shown in Figure 6G. See [35] for more data.

These observations indicate that secondary germ cells develop from OSC in adult human ovaries and form new primordial follicles by assembly with granulosa cell nests.

### Expression of meiotic entry SCP3 protein in adult human and monkey ovaries

In a recent study, Liu and colleagues compared expression of meiotic marker SCP3 in fetal and functionally undefined adult human ovaries [75]. The authors argued that SCP3 protein was not detectable in the tunica albuginea, OSC or in oocytes of primordial follicles in adult ovaries, and hence concluded that no meiotic oocytes are present in ovaries during adulthood. In a subsequent commentary, Tilly and Johnson [76] indicated that the lack of evidence on neo-oogenesis in adult human females is not evidence of its absence, and on the contrary that some data of Liu et al. [75] support the existence of neo-oogenesis in adult women. Subsequently we reported that using the same SCP3 antibody, immune-reactivity with segments of tunica albuginea and OSC, and in some oocytes of primordial follicles in functional adult human and monkey ovaries was detected [71].

Meiotic entry SCP3 protein is expressed in precursor cells of OSC in some segments of tunica albuginea in functional adult human ovaries (arrowhead, Figure 6H) and also in OSC cells of human (panel I) and monkey ovaries [71]. Moreover, SCP3 immunostaining was observed  in the nucleoli of oocytes in some primordial follicles in adult human (arrowhead, panel J) and monkey ovaries (red vs. white arrowhead, panel K). Earlier, Tres had reported that male germ cells exhibit nucleolar SCP3 expression during early stages of meiotic prophase [77]. In addition, an SCP3+ synapsis of two chromosomes was detected in human primordial follicle oocytes (arrowhead, panel L and insert), possibly representing XX chromosomal synapsis, as sex chromosomes start synapsis during early zygotene, before autosomes synapse [77]. Rare SCP3+ oocytes (less than 10%) were detected in midfollicular phase ovaries. The highest expression of SCP3 (10 to 30% of primordial follicle oocytes) was found in postovulatory ovaries during the early luteal phase in younger (up to 38 years of age) women. However, at age 42, postovulatory ovaries showed no SCP3 expression. Virtually no staining of oocytes was observed in three younger women studied during the mid- and late luteal phases, or in polycystic ovaries [71]. Panel M shows SCP3 expression in adult rat testes (positive control). Note SCP3 immunostaining of condensed chromosomes in spermatogonia (red arrowhead) and advanced progression of meiotic division in primary spermatocytes (black arrowhead) in a 2-month-old rat male gonad.

These observations indicate that SCP3 is expressed in adult human and monkey ovaries, confirming that neo-oogenesis occurs in primates during adulthood. Preparation for meiotic activity may have already occurred at the level of tunica albuginea stem cells, and meiotic prophase activity may continue and terminate in oocytes of newly formed follicles. As indicated by Kayisli and Seli: "If proven to occur in human, the implications of de-novo oocyte formation from stem cells would be significant for our understanding of fertility and our approach to its preservation" [78].

### Adult rat ovaries

Studies of human ovaries raise the question as to whether formation of new oocytes exists in other adult mammalian species. We studied ovaries of adult rat females by immunohistochemistry and found migrating ameboid germ cells [36] resembling the migrating germ cells found in adult human ovaries [35], and clusters of dividing germ cells expressing zona pellucida (ZP) proteins in unstained solid epithelial cords. These observations indicated that germ cells, some of which exhibit the ameboid shape, may develop in adult rat ovaries. These cells may originate from the OSC. An alternative site is the ovarian hilar region, which contains sex cords replete with bone morphogenetic protein (BMP) ligands and receptors [79].

Using a double staining immunohistochemistry technique [72], we found that bone marrow derived cells (MDC and T cells) are also involved in triggering germ cell development from the OSC in adult rats. The MDC (not shown) and T cells (black asterisk, Figure 6N) accompanied asymmetric division of OSC giving rise to ZP+ germ cells  (red vs.  yellow asterisk). These descend into the adjacent solid epithelial cord, also described in ovaries of adult guinea pigs [80], a source of granulosa cells under the OSC layer. Large oogonia divided symmetrically (crossing over) in the solid epithelial cords (Figure 6O; see also [81] for mice), and such division was accompanied by MDC (yellow arrowhead, panel P). Blue arrowheads indicate association of solid epithelial cord cells representing primitive granulosa cells. Note that in adult human ovaries symmetric division of emerging germ cells is apparent (Figure 6E) and no dividing oogonia were detected [35], while in adult rat ovaries besides emerging germ cells [36], the new oogonia can also symmetrically divide (panels O and P).

Divided rat oogonia separated, and the resulting oocytes formed new primordial follicles. Monocyte-derived cells also accompanied the growth of primordial follicles. In adult rats lacking OSC after neonatal estrogen treatment, the germ cells indeed originated in the ovarian hilar region (see above) and formed primordial follicles in the juxtaposed (deep) ovarian cortex [72].

These observations indicate that similar pathways of new oocyte development exist in different mammalian species, although there may be variations in the routes of granulosa cells contributing to the formation of new follicles. For example, the availability of epithelial cell cords in adult rats resembles human fetal ovaries [36]. In contrast, OSC in adult human ovaries produce the cord cells which are very similar to some of the granulosa cells. In some areas of the ovary, cords fragment and appear as small 'nests' of epithelial cells. Typically, these epithelial nests (fragmented cords) lie in proximity to primordial follicles [82,83].

Our observations indicate that these primitive granulosa cell nests are descending into the deep cortex where they assemble with vessels to catch circulating oocytes or surround OSC crypts to assemble with migrating germ cells [35]. Hence in adult women, the number of granulosa cell nests determines the number of newly formed follicles, since superfluous new oocytes degenerate in medullary vessels. Accordingly, even if some new oocytes form after the end of the prime reproductive period (PRP; women between menarche and 38+ 2 years of age - reviewed in [35,84]), the lack of developing granulosa cell nests precludes the formation of new follicles. Due to the progressive diminution of the remaining aging follicular pool, menopause occurs. Preliminary termination of either new oocyte or granulosa cell nest formation results in premature ovarian failure [85].

### Bone marrow derived cells and the "storage" vs. a "prime reproductive period" doctrines

In 1923, Edgar Allen [81] introduced a distinction between the "storage" theory, which is based on the opinion that there may never be any increase in the number of oocytes beyond those differentiating during fetal or perinatal ovarian development [86], versus the "continued formation" of oocytes theory, which suggests that oogenesis is maintained throughout the life of mammals [35,57,71,80,81,87].

The currently prevailing "storage" doctrine, as elaborated by Sir Solly Zuckerman and collaborators (reviewed in [88]), is based on the following milestones (assumptions): A) Total number of oocytes declines with age by a simple regression.

B) Oocytes persist in rat ovaries lacking ovarian surface epithelium (i.e. OSC).

C) Oogonia do not persist in adult ovaries.

D) Oogenesis from somatic stem cells is missing.

E) Mitotic division of oogonia is missing.

Regarding assumption (A), there is no significant decline during 20 years of reproductive life, between 18-38 years of age in humans [89]. In addition, Faddy [90] indicated that the pattern of primordial follicle number decline is not exponential, but more bi-exponential corresponding to a 'broken-stick' regression of logged total numbers of follicles against age. Such a model implies an abrupt change in the exponential rate of follicle loss at age 38 years, and is thus rather implausible biologically [90]. The model, however, will be biologically plausible when follicular renewal is considered to act before (slow decay rate during oocyte renewal) but not after 38 years of age (fast decay rate during oocyte storage).

Regarding the argument (B) that OSC are not essential for neo-oogenesis since the oocytes persist in ovaries lacking OSC, we recently demonstrated that in rat ovaries lacking OSC, the oocytes originate by an alternate pathway, from medullary somatic stem cells; primordial follicles are formed in the juxtaposed (deep) ovarian cortex [72].

Assumption (C) is in principle correct, since the oogonia should not persist in adult ovaries, due to the threat of accumulation of genetic anomalies with age. Yet, in adult human females, precursors of germ cells are tunica albuginea stem cells [35], which have a mesenchymal character and are certainly more resistant to environmental threats and to the accumulation of genetic abnormalities with age. Differentiation of OSC from ovarian tunica albuginea precursors is triggered by activated MDC [70].

Regarding point (D), step by step oogenesis and follicular renewal from somatic stem cells have been described in fetal and adult human and adult rat ovaries [35,36,57,72].

Finally, regarding query (E), the mitotic division of newly formed germ cells and oogonia has been described in human and rat ovaries [35,72].

Regarding both the storage and continued oocyte formation paradigms, there appears to now be a consensus that germ cells per se do not persist in adult mammalian ovaries from the fetal/perinatal period. From the view of groups attempting to re-establish the "continued formation" doctrine and search for the origin of new germ cells in adult humans and laboratory rodents [35,57,72,91], there appears to be a consensus that during adulthood the germ cells originate from progenitor cells. Two possible mechanisms for the generation of new oocytes in postnatal mammals have been recently proposed by Joshua Johnson [92].

1) New oocytes are produced via germ stem cells that reside in an extragonadal location, the bone marrow, and are released into the peripheral blood. These progenitors migrate to the ovary, where they may engraft as new oocytes within new follicles [91]. The developmental potential of labeled oocytes after bone marrow transplantation remains unclear [93].

 2) New oocytes are produced by a transformative mechanism. Ovarian bipotential progenitor cells produce both new oocytes and somatic (granulosa) cells within the ovary [35,57,94].

More recently, it has been reported that bone marrow transplantation improves attenuated fertility after low dose chemotherapy in mice, although all newborns were of recipient and not of bone marrow donor origin [95]. Tilly's group introduced in 2004 the idea of the origin of female germ cells in mice from persisting germline stem cells in the ovary [96]. A year later, this was replaced with the idea of the extra ovarian origin of mouse putative germ cells from bone marrow [91]. They also now found the idea on the origin of germ cells from bone marrow untenable, suggesting that bone marrow cells function primarily by reactivating host oogenesis impaired by chemotherapy [95]. However, they did not indicate which bone marrow cells are involved and how and where the new germ cells originate in the recipient. Our studies suggested that the bone marrow derived white blood cells (monocytes and T lymphocytes) accompany the origin of new germ cells from OSC in fetal and adult human and adult rat ovaries, or from medullary stem cells in adult rats lacking OSC (reviewed in [72,84]). Furthermore, activated resident vascular pericytes and bone marrow derived monocytes accompany initiation of follicular growth, selection, and preovulatory maturation of autologous oocytes [57,70,84]. We propose that the lack of activated pericytes and bone marrow derived monocytes committed for the stimulation of follicular growth and maturation of the allogeneic (donor) oocytes may be why the primordial follicles formed from circulating donor germ cells were found to be unable to differentiate and undergo ovulation [91,93,95].

During certain periods of life, however, the storage of oocytes in mammals may occur. Recently, we attempted to establish a harmony between the "storage" and "continued formation" theories by proposing the "prime reproductive period" theory [56,72,84,97] as follows: the "storage" theory pertains to two periods of the life in human females, that is between the termination of fetal oogenesis and puberty or premenarcheal period (about 10 to 12 years), and premenopausal period following the end of the PRP until menopause (also about 10 to 12 years). On the other hand, the "continued formation" theory accounts for the follicular renewal during the PRP (about 25 years, i.e., between menarche and 38+ 2 years of age), and ensures an availability of fresh oocytes for the development of healthy progeny. Since the number of primordial follicles begins to diminish in aging rodents [98], one may consider the relevance of the PRP theory in these species as well.

In conclusion, we are convinced that the neo-oogenesis and follicular renewal during the PRP exists throughout the animal kingdom, including higher vertebrates.

### Vascular pericytes and MDC regulate differentiation and selection of human ovarian follicles

**Figure 7.**
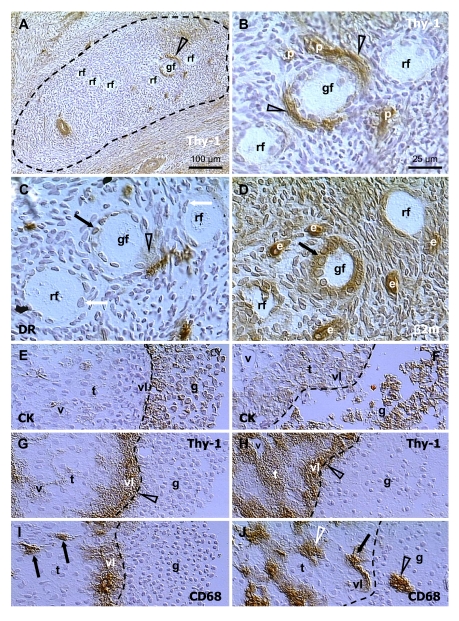
Selection of secondary (**A-D**) and preovulatory (dominant) follicles (**E-F**) in the adult human ovary. Staining for Thy-1, HLA-DR (DR), MHC class I light chain (β2m), cytokeratin 18 (CK)  and CD68 of mature MDC, as indicated in panels. Dashed line in (**A**) indicates an area exhibiting diminution of Thy-1 expression by stromal cells. (**B**), detail from (**A**). (**C**) and (**D**) are semi-parallel sections to (**B**). Dashed line in (**E-J**), follicular basement membrane. rf, resting follicles; gf, growing follicle; p, pericytes; e, endothelial cells; v, microvasculature in theca interna (t); vl, vascular layer adjacent to the follicular basement membrane; g, granulosa layer. Details in text. Adapted from Ref. [70], © Wiley-Blackwell.

Within the adult human ovary, cohorts of primordial follicles occupy distinct areas in the cortex (dashed line, Figure 7A), characterized by diminution of Thy-1 expression in stromal cells [35,70] In these areas stromal cells show enhanced MHC class I expression [57]. Most of the primordial follicles remain in the resting state (rf, Figure 7A), but some show an increase in size and apparent transformation into growing (secondary) follicles (gf) accompanied by increased activity (Thy-1 release) of pericytes (arrowheads, Figure 7B). This could be stimulated by due permissive signals from innervation of follicular vessels (autonomic innervation +, Figure 1), since innervation controls quantity, but not quality of tissues and their structures [32]. Initiation of follicular growth is also associated with an interaction of pericytes (arrowheads, Figure 7B) and activated macrophages (semi-parallel section, Figure 7C). Note HLA-DR+ material, an indicator of activated MDC [99], secreted near granulosa cells and oocyte (arrowhead, Figure 7C), and accumulating in the nuclear envelope of granulosa cells (black vs. white arrows). Figure 7D (semiparallel section to B and C) shows strong MHC class I expression, an indicator of epithelial cell differentiation (see Figure 2F), and the cuboidal shape of granulosa cells, which accompanies this process.

Usually only one dominant follicle is selected for ovulation during the mid follicular phase of each menstrual cycle in the human ovary. This process of follicular selection still remains an unresolved puzzle. Premature stimulation with gonadotropins results in multiple ovulations, suggesting that more than one large antral follicle in the cohort developing up to the middle of the follicular phase is capable of ovulating. Hence, under normal conditions, there seems to be a competition among growing follicles themselves in an attempt to reach the mature state and suppress the development of others. In contrast with this traditional view, our data indicate that the follicles showing the most advanced development during selection are not the dominant follicles. A critical role in the process of dominant follicle selection appears to belong to the theca interna compartment [29,100].

In antral follicles of mammalian ovaries, including humans, two clear cut zones in theca interna were detected. About one-third of the cells corresponding to a more internal region (inner or vascular layer) were not stained with luteinizing hormone receptor, 3-beta-hydroxysteroid dehydrogenase, and P450-17alpha-hydroxylase antibodies. This contrasted with the remaining two-thirds of cells corresponding to the external regions (outer or steroidogenic layer), which were strongly labeled [101-103]. In dominant follicles, the inner vascular layer of theca interna contains vascular pericytes secreting Thy-1 differentiation protein among differentiating granulosa cells [27,29].

Figure 7E shows cytokeratin staining of a human dominant follicle in mid-follicular phase with multiple granulosa cell layers (g) adjacent to the basement membrane (dashed line). Under the follicular membrane is a vascular theca interna layer (vl) surrounded by a steroidogenic theca interna layer (t) with narrow vessels (v). Staining for Thy-1 (Figure 7G) shows that a high activity of Thy-1 pericytes is restricted to the vascular layer. The MDC releasing CD68 (Figure 7I) are absent from the steroidogenic layer (arrows), but abundant in the vascular layer.

Large antral follicles undergoing atresia in the same ovary show detachment of granulosa cells from the basement membrane (Figure 7F). This is accompanied by activation of pericytes in the steroidogenic layer (white t, Figure 7H) and dilatation of vascular lumina (v; compare panels F and H vs. E and G). In addition, the MDC in the steroidogenic layer become highly activated (white arrowhead, Figure 7J), but those in the vascular layer show low or no CD68 release (arrow). Instead, the MDC from the vascular layer invade among granulosa cells (black arrowhead) [70].

Since regulation of follicular selection involves autonomic innervation [59,60], we suggest that activation of pericytes in the steroidogenic thecal layer of follicles undergoing atresia is caused by permissive neuronal signals. On the other hand, retardation of pericyte activity in a dominant follicle steroidogenic thecal layer during selection is caused by a lack of such signals (autonomic innervation -, Figure 1).

### Novel aspects of follicular selection

Follicles are selected twice during their development (secondary from primordial and preovulatory from antral follicles), but the consequences for the remaining follicles are different. First, during basal growth, secondary follicles are selected from primordial follicles under the control of growth factors of paracrine origin. Unselected primordial follicles remain in the resting state. The selection of secondary follicles is associated with activation of pericytes in adjacent micro-vasculature, possibly due to permissive signals from autonomic innervation, which is involved in the regulation of quantitative aspects (amounts) of specific cells and structures in tissues from early periods of life [32,104]. This also causes an activation of perivascular MDC. Hence, during growth initiation, the selected follicles are stimulated in further development.

After attaining the antral stage, follicles become gonadotropin dependent and immature granulosa cells can be affected by thecal androgens [105]. Hence, premature acceleration of theca interna steroidogenic layer development may cause follicular atresia by thecal androgens via alteration of immature granulosa cells lacking aromatase. This is associated with conversion of follicular MDC into phagocytes infiltrating the follicular antrum. We show that during selection of pre-ovulatory follicle, the pericytes in steroidogenic layer of theca interna in non-dominant follicles are highly activated and accompanied by activated MDC. In contrast, non-activated MDC are present in the vascular layer adjacent to the follicular basement membrane, and invade the granulosa layer of non-dominant follicles. Hence, it appears that the dominant follicle is selected by a process of temporary retardation of steroidogenic thecal differentiation, possibly by a negative influence of autonomic innervation on thecal pericytes. Extracts of the superior ovarian nerve have been shown to inhibit thecal cell androstenedione production [106,107].

Once the dominant follicle matures into the preovulatory stage, with the ability of mature granulosa cells to convert androgens into estrogens [105], pericytes and MDC in both steroidogenic and vascular layers of the dominant follicle show high activity [29,57]. Taken together, acceleration of steroidogenic thecal layer development during follicular selection results in premature androgen production causing detachment of immature granulosa cells from the follicular basement membrane, invasion of macrophages into the follicular antrum, and progression of atresia of non-dominant follicles [70].

### Corpus luteum

The CL of the menstrual cycle has the shortest lifespan of any tissue structure in the mammalian body. In women, its function ceases after two weeks, followed by transformation into the amorphous corpus albicans. The association of various types of immune cells with the CL during its development and regression indicates that the immune system is involved in CL management. The absence of the CL during early ontogeny, including immune adaptation, suggest that the CL could be viewed by the immune system as a graft [17]. Although the ovary is densely innervated, with autonomic nerves associated with thecal vessels of all follicles regardless of the stage of development, the luteal vessels lack autonomic innervation [108]. Another feature of the CL is that, in contrast to some other tissues, such as the liver, it is unable to regenerate. Moreover, during pregnancy, the CL can survive and function for an extended period. This longevity is associated with a change in behavior of luteal mesenchymal and immune cells. Thus the CL is a unique model for the study of TCS mediated mesenchymal-epithelial interactions without influence of innervation.

**Figure 8.**
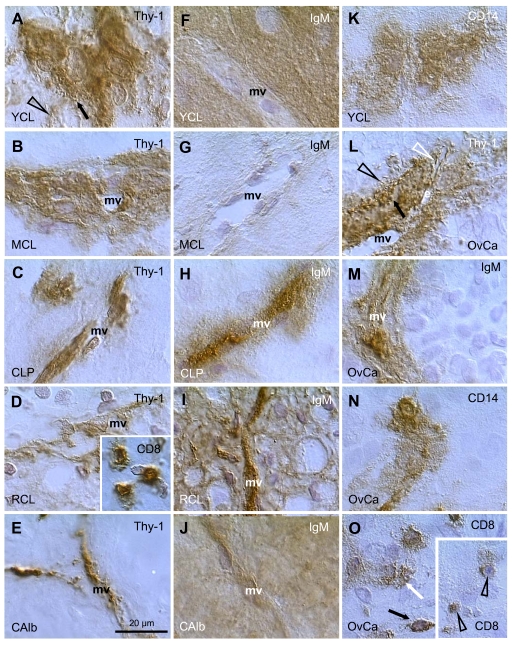
Staining for Thy-1, IgM, CD8, and CD14, as indicated in panels, in human corpora lutea and ovarian adenocarcinomas (OvCa). YCL, young CL; MCL, mature CL; CLP, CL of pregnancy; RCL, regressing CL (subsequent follicular phase); CAlb, corpus albicans. mv, microvasculature. Scale bar in E applies to panels A-O, including insets. Details in text. Adapted from Ref. [109], © Elsevier.

Figure 8A shows a young CL (2 days after the LH peak) with high activity of Thy-1 pericytes, characterized by secretion of intercellular vesicles (arrow) which are converted into empty "spikes" (arrowhead). A mature CL (5 days post ovulation) shows partial diminution of pericyte activity (Figure 8B). In the CL of pregnancy (3rd month) the pericytes persist in an inactive state (Figure 8C). Regressing CL (early follicular phase of the next cycle) shows regression of pericytes (Figure 8D) and infiltration by T cells (inset). This is accompanied by IgG binding to luteal cells (not shown). Similar features were observed during CL regression at the end of pregnancy. In the corpus albicans no luteal cells are present and remnants of pericytes accompany regressing microvasculature (Figure 8E).

The IgM distribution is shown in Figure 8F to J. When compared to IgG, IgM is expressed phylogenetically (sharks) and ontogenetically (immature B cells) much earlier [99]. Accordingly, IgM shows binding to tissue cells at various stages of their differentiation. In the stratified epithelium IgM binds to the young (parabasal) cells (IgM1), isolated mature and all aging (top of interstitial layer) cells (IgM2), and a layer of the most superficial cells (IgM3) [3,51] - see also above. The IgM also binds to endothelial cells, depending on the stage of differentiation of tissue cells.

Figure 8F shows strong IgM binding to the granulosa lutein cells in the young CL. Note a lack of binding to the endothelium of microvasculature (mv). In the mature CL no IgM binding is apparent to either granulosa lutein or endothelial cells (Figure 8G). The CL of pregnancy shows no IgM binding to granulosa lutein cells, but strong binding to the vascular endothelial cells extended toward the pericytes (Figure 8H). In regressing CL (Figure 8I), IgM binds to both regressing luteal cells and vascular components. In the corpus albicans, IgM binds to the amorphous structure and to residual microvasculature (Figure 8J).

Perivascular primitive CD14 MDC show high activity (secretion of CD14 material into the intercellular space) in the young CL (Figure 8K). This is accompanied by secretion of CD68 and HLA-DR by MDC. The activity of MDC diminishes in the mature and aging CL, where the MDC show a conversion into dendritic cells. Subsequently, from the beginning of the next menstrual cycle, the luteal cells show strong expression of various MDC and leukocyte markers, including CD14, CD68, HLA-DR, leukocyte-common antigen, and CD4 of MDC and of helper T cells [17].

These observations indicate that the TCS components vary with CL development, preservation during pregnancy, and regression. High activity of vascular pericytes and primitive MDC is characteristic for the CL development, and T cells and dendritic cells accompany CL regression, resembling graft rejection.

### OVARIAN CANCERS

Ovarian cancers represent a wide variety of cell types with variable metastatic potential. The most common types are adenocarcinomas, often expanding into the peritoneal cavity and metastasizing to the omentum. Depending on the location (primary vs. metastatic), and stage of differentiation (poor, moderate, well differentiated), the activity of stromal and intraepithelial mesenchymal cells in ovarian carcinomas varies. Figure 8L-O, shows several examples of mesenchymal cell activity in various ovarian adenocarcinomas in addition to those reported earlier [108,109].

Figure 8L shows high activity of Thy-1 pericytes in a poorly differentiated ovarian adenocarcinoma. Note secretion of Thy-1 vesicles (arrow), the presence of empty spikes among malignant cells (black arrowhead) and in the sprout of endothelial cells (white arrowhead). Such activity is similar to that seen in the young CL (see Figure 8A). However, pericytes in well differentiated adenocarcinomas show low or no activity, similar to that observed in the persisting CL of pregnancy.

IgM binding is restricted to the microvasculature (Figure 8M), and none of the 20 adenocarcinomas investigated showed IgM binding to proliferating (Ki67+) or differentiating malignant cells. This situation is also similar to the persisting CL of pregnancy.

The most common feature seen in ovarian cancers was the high activity of MDC. Figure 8N shows secretion of CD14 from immature MDC into the malignant epithelium (see also young CL in Figure 8K). Similar activity was observed in CD68 and HLA-DR monocyte-derived cells [109]. A proportion of adenocarcinomas (9/20) showed infiltration of malignant cells by T lymphocytes. Panel O shows normal T cells within the malignant stroma (black arrow). T cells which enter differentiating malignant epithelium release CD8 material among malignant cells (white arrow). Deeper within the tumor, the T cells become smaller and exhibit low CD8 expression (arrowheads in inset), features characteristic of their apoptotic fragmentation [4,108].

### MDC in ovarian epithelial inclusion cysts and pro-inflammatory cytokines in ovarian cancers

**Figure 9.**
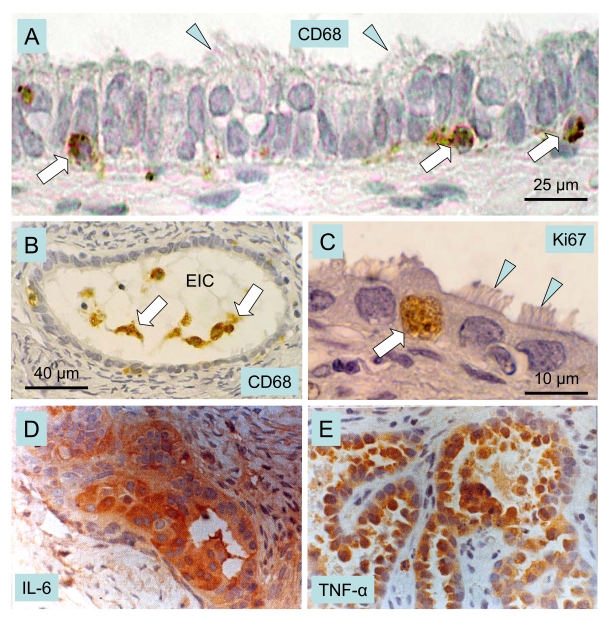
Macrophages, cytokines and ovarian cancer. Epithelial inclusion cysts (EIC), showing infiltration of the cyst wall (**A**) and lumen (**B**) by CD68 positive MDC (arrows), ciliated cells (arrowheads), and Ki67 positive (arrow in **C**) proliferating cells. Immunohistochemical staining of cancerous ovarian tissues for IL-6. (**D**) and TNF-alpha (**E**) (x400). A-C adapted from Ref. [111], © Elsevier, and D and E from Ref. [113], © John Libbey Eurotext Ltd.

Epithelial inclusion cysts (EICs), are formed by trapping of ovarian surface epithelium (OSE) cells within the ovarian stroma during ovulation wound repair or ovarian surface inflammatory processes. It is widely accepted that EICs constitute a preferential site of ovarian carcinogenesis. OSE cells in EICs undergo Müllerian metaplasia and acquire the architectural and functional characteristics of the epithelia of Müllerian duct derivatives, such as Fallopian tube, endometrium or endocervix. Tubal metaplasia, the most common differentiation pathway in EICs, is characterized by the appearance of secretory and ciliated cells (arrowheads, Figure 9A and C) and expression of specific genes such as CA125 and oviductal glycoprotein [110]. Notably, differentiation of OSE cells in EICs through Müllerian pathways is associated with the presence of monocyte derived CD68 positive cells (MDC) that infiltrate the cyst wall and accumulate in the cyst lumen (arrows, Figure 9A and B) [111]. MDC are a source of active cytokines that could reach bioactive concentrations in the confined space of the EICs, thus affecting the differentiation and proliferative activity of epithelial cells (Figure 9C) contributing to the initial stages of OSE cell transformation.

The levels of pro-inflammatory cytokines IL-1 alpha, IL-1 beta, IL-6 and TNF-alpha were higher in ovarian cancerous tissues than in normal specimens [112,113]. The higher levels of these factors were detected mainly in epithelial cells of the tumor than in the surrounding stromal cells (Figure 9D and E).

Altogether, there might be novel strategies for the targeting activity of MDC in "tissue control units" associated with cancer [109]. Figure 8N shows association of primitive CD14 MDC with growing malignant cells. CD14 is a lipopolysaccharide receptor [114], which inhibits activation of NK cells capable of recognizing and killing tumor cells [115]. It is possible that temporary targeting of CD14 MDC, e.g. by CD14 antibody [109], small interfering (si)RNA [116], or other approaches targeting tissue macrophages, may result in the regression of unstable (lack of autonomic innervation) "tissue control units" in malignant tissues, leading to activation of NK cells, and subsequent regression of malignant stroma and elimination of tumor cells.

These data indicate that malignant growth is associated with enhanced activity of mesenchymal cells, and tissue macrophages in particular. Novel approaches to cancer prevention and control may depend on a better understanding of the mechanisms by which tissue macrophages promote growth of tumor cells. In addition, studies of events accompanying regression of luteal tissue may be of importance for better understanding on how the regression of vascular components results in ultimate regression of epithelial/parenchymal and possibly malignant tissues [70].

### IMMUNE ADAPTATION AND THE DETERMINATION OF FUNCTIONAL TISSUE LIFESPAN

During immune adaptation (through the end of the second trimester of intrauterine life in humans [99]), differentiating tissues are recognized by the developing lymphoid (immune) system as self [117-119]. However, depending on the time point at which a certain tissue arises during immune adaptation, cellular memory can determine how long MDC and T cell support will persist. In the ovary, these cells influence formation of new germ and granulosa cells and differentiation of primordial follicles [36,57].

**Figure 10.**
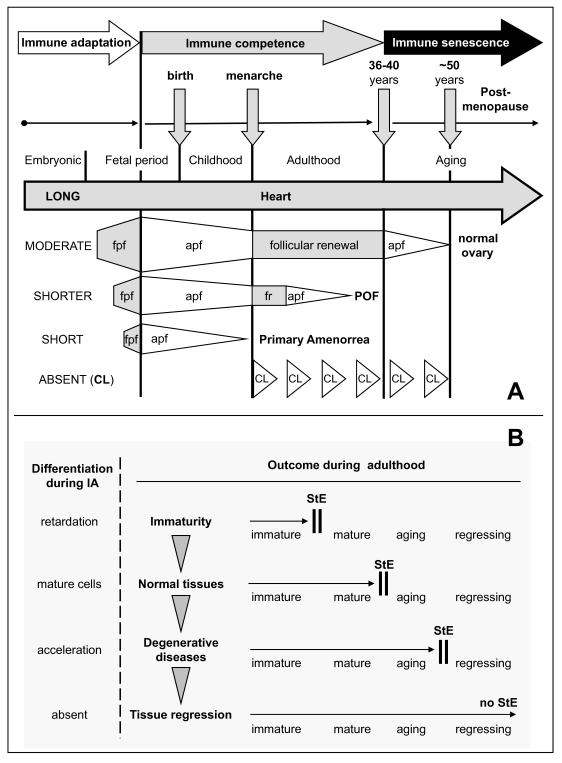
Immune adaptation and TCS "stop effect." (**A**) Immune adaptation (IA) and tissue longevity. The heart differentiates from early stages of ontogeny (LONG IA) and functions throughout life. The ovary differentiates later (MODERATE IA), and its normal function is limited by follicular renewal (until 35-40 years of age). Aging primordial follicles (apf) persist until exhausted (physiologic menopause). SHORTER period of ovarian development during IA causes earlier termination of follicular renewal during adulthood and results in POF. SHORT period of ovarian development during IA causes no follicular renewal and results in primary amenorrhea. Absence of corpora lutea (CL) during immune adaptation causes their cyclic degeneration, except during pregnancy, which is accompanied by immune suppression. fpf, fetal primordial follicles; fr, follicular renewal; POF, premature ovarian failure; CL, corpus luteum. Adapted from Ref. [31]. (**B**) Stages of cell differentiation during immune adaptation (left) sets TCS "stop effect" (StE) for tissue physiology and pathology during adulthood. Arrowheads indicate a tendency to StE "shifts" with age. Adapted from Ref. [3,30,33,108].

In normal adult individuals, the first organ affected by aging is the thymus [120], and the next are the ovaries [121,122]. There is a correlation between the period at which an organ is present during early ontogeny and its functional longevity. For instance, the heart, which differentiates very early, can function in humans for over one hundred years. In contrast, the ovaries, which differentiate later, do not function for more than half that time (Figure 10A). We have proposed that the later the differentiation of certain tissues occurs during early ontogeny, the earlier its function expires during adulthood [31]. Ovarian development is influenced by mesenchymal-epithelial interactions which accompany the emergence of germ cells and follicular growth [4,57,70]. Uncommitted MDC may first recognize and memorize the character of OSC, which differentiate from urogenital coelomic epithelium populated by primordial germ cells. In the fetal ovary, presumptive memory cells reside in the rete ovarii, and uncommitted MDC and T cells migrate through rete channels toward the ovarian surface and participate in the development of germ cells from the OSC [36]. Similar interaction of immune cells with OSC was described in the ovaries of adult women [57]. During adulthood, however, no rete is present in ovaries, so the memory cells may reside in the lymphoid tissues, the source of antigen-committed immunocytes [99]. The immune system shows a significant functional decrease between 35 and 40 years of age in women [123], and concomitantly ovarian follicular renewal wanes [35].

Premature ovarian failure (POF) could be caused by delayed ovarian development during immune adaptation (SHORTER, Figure 10A), by earlier termination of immune adaptation, or by cytotoxic chemotherapy affecting both the existing pool of primordial follicles and the OSC committed bone marrow-derived cells (T cells in particular) required for the emergence of new secondary germ cells and hence for follicular renewal. Patients with POF have been found to have abnormalities in the function of circulating monocytes, activated lymphocytes, and NK cells, and exhibit other immune abnormalities [124-126], suggesting a relationship between immune system and POF.

### Thymus and reproduction

The thymus plays an important role in the immune system, and it has been suggested that thymic peptides participate in determining the reproductive lifespan of females [127,128]. The relationship between age-associated thymic involution and diminution of ovarian function is evidenced by the alteration of ovarian function in neonatally thymectomized mice [129]. In congenitally athymic (nude) mice, follicular loss is first evident at 2 months of age, specifically due to a reduction in the numbers of primordial follicles. The first ovulation is delayed until two and a half months of age, compared to one and half months in normal mice. By four months, an overall reduction in all fractions of the follicle population occurs in nude mice, and ovulation ceases [130].

### THE TISSUE CONTROL SYSTEM AND A "STOP-EFFECT" OF MDC THEORY

By the end of immune adaptation in early ontogeny, the MDC are proposed to encounter the most differentiated cells in a specific tissue, and prevent them from differentiating beyond the encoded state during adulthood by the so called "stop effect." The power of this "stop effect" may reside in the inability of monocyte-derived cells to stimulate differentiation of tissue cells beyond the encoded stage [3]. Retardation or acceleration of differentiation during immune adaptation may cause a permanent alteration of tissue function. If the ability of monocytes to preserve tissue cells in a functional state declines with age, a functional decline would ensue, leading to menopause and degenerative diseases.

In large mammals including primates immune adaptation ceases during intrauterine life, while in laboratory rodents (rats and mice) immune adaptation continues for several postnatal days, ending about one week after birth [99]. Estrogens given to neonatal rats or mice inhibit ovarian development, and during adulthood females show persisting ovarian immaturity characterized by a retardation of follicular development [30] despite normal serum levels of gonadotropins [131,132]. This indicates that suppression of early ovarian development results in persisting ovarian immaturity, which resembles POF associated with the gonadotropin resistance of ovarian follicles. Injection of estrogens in neonatal mice (days 0-3) caused permanent anovulation, but mice injected later (days 3-6; closer to the end of immune adaptation) showed resumption of ovulatory cycles after initial anovulation [133]. Hence, persisting ovarian immaturity can result in a delay of normal ovarian function. Since the incidence of degenerative diseases increases with age, one may expect the "stop-effect" to shift with age (arrowheads, Figure 10B). This could explain why an immature ovary may switch to a functioning ovary.

On the other hand, injection of androgens causes premature ovarian aging which persists during adulthood. However, androgen induced anovulation may be prevented by neonatal injection of a thymic cell suspension from immunocompetent prepubertal normal female donors, unless the animal donors did not complete immune adaptation [134,135]. This suggests that certain thymic cells (thymocytes, or thymic MDC) of normal immunocompetent females carry information about appropriate differentiation of ovarian structures, and this information can be transferred to immunologically immature neonatal rats. Hence the state of tissue differentiation during immune adaptation determines tissue function in adult individuals.

When a lower dose of androgens is injected during immune adaptation, the rats exhibit a so-called delayed anovulatory syndrome. Ovaries exhibit the onset of normal function after puberty (~40 days of age), but premature aging of the ovary occurs between 60-100 days [136]. This delayed manifestation of ovarian dysfunction resembles human POF with secondary amenorrhea as well as some human degenerative diseases with an autoimmune character. The latter similarly occur after a shorter (juvenile diabetes mellitus) or longer (Alzheimer's disease) period of normal tissue function.

A simplified view of the TCS theory of the regulation of tissue function via the "stop effect" (StE) is depicted in Figure 10B (see also [4,17,34]). In normal tissues, the mature cells are present during immune adaptation, and the tissue-specific cells are "parked" in the mature state during adulthood. Retardation of cell differentiation during adaptation results in persisting immaturity (POF with primary amenorrhea) and acceleration in premature aging (POF with secondary amenorrhea, degenerative diseases). If the tissue is absent during adaptation (e.g. corpus luteum), it will be handled immunologically as a "graft."

### IMMUNE PHYSIOLOGY AND REGENERATIVE MEDICINE

### Totipotency of ovarian stem cells *in vitro*

 Ovarian stem cells exhibit a totipotent character resembling embryonic stem cells, since they are able to differentiate *in vitro* into oocytes, parthenogenetic embryos, and neural/neuronal cell types, [94,137,138] and OSC cultures exhibit markers of embryonic stem cells [139,140].

**Figure 11.**
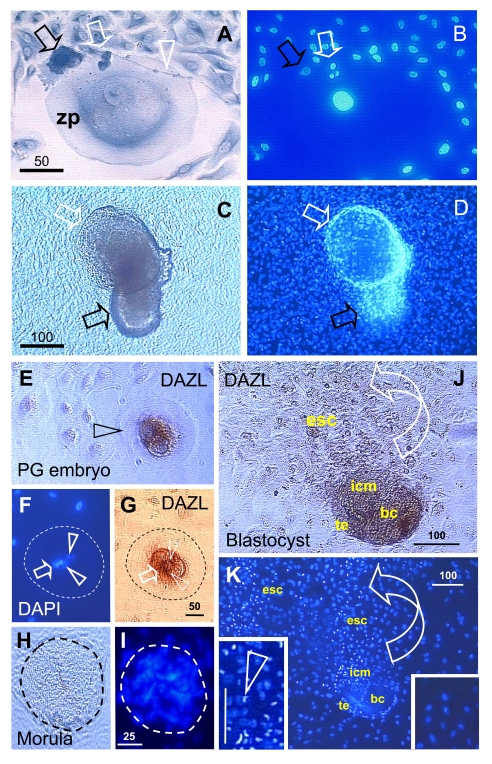
Oocyte and parthenote development *in vitro*. (**A**) The oocyte development in OSC culture is accompanied by a satellite (black arrow) and neuronal (white arrow) cells. White arrowhead indicates neuronal extension. (**B**) DAPI staining of (**A**). (**C**) The parthenote shows a blastocoele (white arrow) and inner cell mass (black arrow). (**D**) DAPI staining of (**C**). Four cell embryo. (**E - G**) and morula (**H** and **I**). **J **and **K **panels show a blastocyst consisting of blastocoele (bc), trophectoderm (te), and inner cell mass (icm) releasing ESC (esc). Left insert in panel **K **shows enhanced DAPI staining of dividing ESC vs. low DAPI staining of other cells in the culture (right insert). Details in text. Adapted in part from Ref. [137], © Cambridge Journals.

Advanced differentiation of oocytes *in vitro* produced oocytes of 200 μm diameter (Figure 11A) - note the wide ZP and compare with the small size of surrounding cells. The oocyte was accompanied by satellite cells (black arrow) substituting for granulosa cells in providing additional resources (organelles) needed by the developing oocyte [141]. Also involved were neuronal type cells (white arrow) with an extension (arrowhead) expanding over the oocyte. Panel B shows DAPI staining - note a large oocyte nucleus. Some oocytes differentiated into parthenogenetic embryos with extensively developed blastocoels (white arrows, panels C and D) and inner cell mass (black arrows).

Deleted azoospermia like (DAZL) protein was strongly expressed in early parthenotes (arrowhead, Figure 11E), at the four cell stage (panels F and G). Resulting morulae (panels H and I; no immunohistochemistry) can develop into blastocysts that show production of DAZL+ embryonic stem cells (ESC) from the embryonic inner cell mass into the culture (arched arrow, panel J). The inner cell mass and the released ESC are mitotically active as compared to the other cells lacking DAZL expression and pronounced DAPI staining (left vs. right inset, panel K) [97,137].

These observations indicate that OSC cultures could be a source of oocytes for the treatment of female ovarian infertility, and can also produce ESC for the purposes of autologous regenerative medicine.

### Epithelial to neural/neuronal transition is triggered by a mixture of sex steroids

Within the field of regenerative medicine of neurodegenerative and traumatic neurologic diseases, there is considerable interest in cellular therapy, such as grafting of neural stem cells (NSC) into the CNS in order to induce neuronal renewal and repair of degenerative, traumatic or ischemic defects. Neural stem cells can be isolated from the neonatal or adult CNS and propagated *in vitro* in the presence of mitogenic growth factors prior to use [142,143]. Alternative sources of NSC are ESC, umbilical cord blood, amniotic epithelial cells, bone marrow stem cells, and mobilized peripheral blood CD133+ cells [144-147]. After several passages, these cells can be transdifferentiated into NSC either by fibroblast growth factor-1, 12-otetradecanoylphorbol-13-acetate (protein kinase C activator), isobutyl-methylxanthine (a non-specific inhibitor of phospho-diesterases), and forskolin (protein kinase A activator), or by all-trans-retinoic acid and 2-mercaptoethanol [148]. These substances are not suitable for treatment *in vivo*, however. Previous work from our laboratory demonstrated that occasionally, neuronal cells spontaneously appear in cultures of human ovarian epithelial stem cells [94].

An alternative to the use of organ-tissue specific stem cells for functional grafting to particular sites (topical therapy) could be a "systemic regenerative treatment" with utilization of common drugs with a low molecular weight. Sex steroids may have the potential to stimulate the proliferation and differentiation of existing NSC. They easily pass the blood-brain barrier and can bind to abundant sex steroid receptors in the brain areas important for the regulation of emotions, cognition, and behavior [149]. However it still remains to be determined whether utilization of individual sex steroids alone might be efficient in prevention or treatment of neurodegenerative diseases and traumatic neurologic injuries. To address this question, we studied the effects of sex steroids on the transdifferentiation of totipotent human ovarian epithelial stem cells and porcine granulosa cells into NSC and neuronal cells [138].

**Figure 12.**
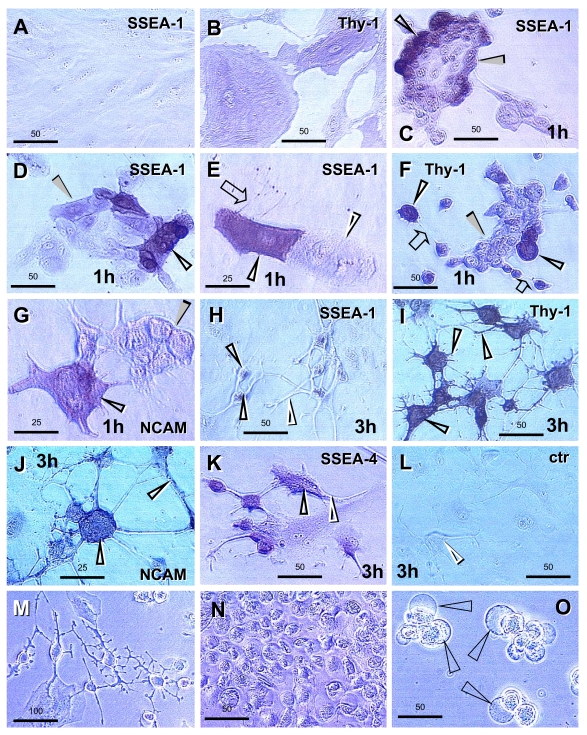
Human ovarian epithelial stem cell cultures (representative images from four experiments): untreated (**A**    and **B**); pre-treated for 1 day with E2 and 1h after TP+PG treatment (**C**-**G**); pre-treated for 1 day with E2 and 3h after TS+PG treatment (**H**-**O**). Lack of SSEA-1 expression (**A**) and moderate Thy-1 expression by some epithelial cells (**B**). SSEA-1 is strongly expressed in some small cells resembling stem cells (black vs. white arrowheads, **C** and **D**) and one of the cells originating by asymmetric division (**E**). Similar cells show strong expression of Thy-1 (**F**). The NCAM expression was also detected in some cells (**G**). Two hours later, the cells reached neuronal morphology and exhibited SSEA-1 expression in the cell bodies but not extending processes (**H**, black vs. white arrowheads), Thy-1 and NCAM expression in both (**I** and **J**, black arrowheads), and SSEA-4 expression slightly exceeding that of SSEA-1 (**K** vs. **H**). No staining was observed in the immunohistochemistry control (**L**). Panels **M-O** show phase contrast microscopy with neuronal and epithelial cells (**M**), floating numerous putative NSC (**N**), and putative NSC exhibiting bubble type anchors (arrowheads, **O**). Numbers above bars indicate microns. For details see text. Adapted from Ref. [138], © Landes Bioscience.

Human ovarian epithelial stem cells differentiated into large epithelial cells lacking expression of stage specific embryonic antigen (SSEA) -1 (Figure 12A) and neural cell adhesion moleculae (NCAM; not shown). A few epithelial cells in untreated cultures showed moderate staining for Thy-1 (panel B) and similar expression of SSEA-4 (not shown). Addition of individual gonadotropins, EGF, or sex steroids alone showed no change in either cell morphology or immune-histochemical staining. No changes were observed in control cultures including those with the sex steroid vehicle.

On the other hand, utilization of testosterone (TS) mixed with progesterone (PG) one day after estradiol (E2) pretreatment produced a marked effect after one hour. There was transdifferentiation of epithelial cell clusters into small cells, a portion of which strongly expressed SSEA-1 glycoconjugate of NSC and precursor cells [150] and others were less densely SSEA-1 positive (black vs. solid grey arrowheads, panels C and D). An asymmetric division resulting in SSEA-1+ and SSEA-1- daughter cells is shown in panel E; note stained early extensions (arrow) associated with the SSEA-1+ cell. Many of the nascent small cells strongly expressed Thy-1, a GPI-anchored protein abundantly expressed by neurons [151]. Particularly strong expression was apparent in cells detaching from the moderately stained cluster of cells (black vs. solid grey arrowheads, panel F). The detached cells, however, still showed connections (arrows) with the cell cluster. More distant cells exhibited development of Thy-1+ extending processes (left black arrowhead), suggesting early stages of neuronal differentiation. Some larger cells with developed extensions strongly expressed NCAM (black arrowhead, panel G), which is characteristic of later stages of neuronal differentiation [150]. Antibody to NCAM is used for the isolation of human ESC-derived neurons [150]. Less developed (smaller) cells in the cluster showed moderate NCAM expression only (solid grey arrowhead).

Three hours after the TS+PG mixture following E2 pretreatment (panels H-O), the cells showed an advanced neuronal morphology with mutually connected extending processes, suggesting what we term "brain in vitro" features. Stage-specific embryonic antigen-1 expression was limited to neuronal cell bodies, and their extending processes were unstained (black vs. white arrowheads, panel H). The neuronal cells strongly expressed Thy-1 glycoprotein in both the cell bodies and extending processes (black arrowheads, panel I), and the same applied for NCAM expression (panel J). Stage-specific embryonic antigen-4 is commonly used as a cell surface marker to identify pluripotent human ESC and expressing cells enriched in the neural stem/progenitor cell fraction [152]. It was strongly expressed in neuronal cell bodies but virtually absent in extending processes (black vs. white arrowhead, panel K). Control immunohistochemistry produced no staining of neuronal (white arrowhead, panel L) or other cell types. In phase contrast observations, the neuronal phenotype cells were shown to interact with remaining epithelial cells (panel M). Large numbers of putative NSC detached from the bottom of the flasks and were found floating in the center of the wells (panel N). These cells showed bubble type anchoring extensions (arrowheads, panel O), which apparently serve to attach seeded non-neuronal cells [138]. Since such putative NSC did not attach again, they may be ready for homing where needed.

In summary, OSC cultures could produce oocytes suitable for *in vitro* fertilization [153], and the treatment can be complemented by implantation of fertilized oocytes in the uterus regardless of the type of ovarian failure. However, regenerative medicine for neurodegenerative and traumatic neurologic diseases depends on local TCS interactions in the given tissue in the particular individual. For instance local or systemic implantation of NSC in Alzheimer's disease does not preclude that such cells will differentiate into mature neurons, if the brain lacks neurosteroids and MDC-derived microglia, supposed to be required for such a process. Moreover, even when such substances and cells are available, the differentiating neurons may not be preserved in a functional state and may continue to degenerate due to the lack of a proper "stop-effect" (see Figure 10B). Proper conditions can be, however, expected in the traumatic neurologic diseases, where regenerative medicine or systemic regenerative treatment may result in a more favorable outcome. Recently, NSC derived from human ESC have been shown to engraft functionally in an experimental mouse model with an improvement of sensimotor function of the stroke-disabled forelimb [154].

## Concluding remarks

Altogether, available data indicate that in addition to its classically defined role in host defense via immune surveillance, the immune system is integral to the differentiation and maintenance of normal tissues via immune physiology. Further study of the TCS, including its involvement in CL development and regression, may also help us understand mechanisms of malignancy and metastasis, and their treatment and prevention. Better understanding of TCS involvement in the preservation of functional tissue longevity via the "stop effect" of MDC could assist in the meaningful utilization of regenerative medicine and systemic regenerative treatment.

## Acknowledgment

This work was supported by the Physicians' Medical Education and Research Foundation and University of Tennessee Research Foundation awards to AB and MRC.
